# A sex skew in life-history research: the problem of missing males

**DOI:** 10.1098/rspb.2022.1117

**Published:** 2022-07-27

**Authors:** C. Ruth Archer, Maria Paniw, Regina Vega-Trejo, Irem Sepil

**Affiliations:** ^1^ Institute of Evolutionary Ecology and Conservation Genomics, University of Ulm Albert-Einstein-Allee 11, 89081 Ulm, Germany; ^2^ Department of Conservation Biology, Estación Biológica de Doñana (EBD-CSIC), Seville 41001, Spain; ^3^ Department of Evolutionary Biology and Environmental Studies, University of Zurich, Zurich, Switzerland; ^4^ Department of Zoology, University of Oxford, Oxford, UK

**Keywords:** antagonistic pleiotropy, demography, life-history strategies, sexual selection

## Abstract

Life-history strategies are diverse. While understanding this diversity is a fundamental aim of evolutionary biology and biodemography, life-history data for some traits—in particular, age-dependent reproductive investment—are biased towards females. While other authors have highlighted this sex skew, the general scale of this bias has not been quantified and its impact on our understanding of evolutionary ecology has not been discussed. This review summarizes why the sexes can evolve different life-history strategies. The scale of the sex skew is then discussed and its magnitude compared between taxonomic groups, laboratory and field studies, and through time. We discuss the consequences of this sex skew for evolutionary and ecological research. In particular, this sex bias means that we cannot test some core evolutionary theory. Additionally, this skew could obscure or drive trends in data and hinder our ability to develop effective conservation strategies. We finally highlight some ways through which this skew could be addressed to help us better understand broad patterns in life-history strategies.

## 1. Background

A new-born Greenland shark may live for more than 300 years [1], but a newly eclosed adult mayfly will seldom live longer than 3 days [2]. Female opossums give birth approximately 13 days after conception [3], while deep-sea octopus mothers guard their eggs for over 4 years before young emerge [4]. Humans are more likely to die and less likely to reproduce as they age, but the opposite is true of desert tortoises, whose survival and fecundity rise with age [5]. These examples illustrate diverse solutions to a universal problem: how should individuals invest in growth, reproduction and somatic maintenance over their lives or otherwise schedule their life histories?

Understanding variation in life-history strategies is a fundamental aim of evolutionary biology [6], population ecology and basic ageing research [5]. From an ecological perspective, characterizing life histories is a key step in predicting which species may benefit from environmental change (e.g. become invasive [7]) or risk extinction [8,9]. Despite this importance, our understanding of how individuals schedule their reproductive investment, and in turn, manage trade-offs involving the costs of reproduction, seems to be based primarily on females [10]. This bias is a concern because the trade-off between reproduction and lifespan is central to life-history theory and evolutionary theories of ageing [6,11,12], and reproductive scheduling is a key axis of life-history variation across species [13]. While other authors have flagged the data paucity for male reproductive schedules (e.g. [11,14,15]), to the best of our knowledge, the general scale of the problem has not been quantified and its impact on our understanding of evolutionary ecology has not been fully discussed.

This review summarizes key reasons why life histories can differ across the sexes. Next, we use open-access demographic data available for tetrapods to demonstrate the existence of a sex skew and quantify its magnitude. We use these data to test our predictions that the sex skew will be more pronounced for reproductive than survival traits given that survival can usually be measured readily in both sexes, but reproduction is typically harder to measure in males than females. We also predict that the skew in availability of reproductive data will be more pronounced in taxonomic classes where parental care tends to be performed by females (i.e. mammals) compared to classes where both sexes tend to provide care (i.e. birds) or where care is largely absent (i.e. reptiles and amphibians) [16] due to the relative ease of assigning maternity versus paternity in each of these systems of parental care. Additionally, we use a semi-quantitative literature review to test the hypothesis that the sex skew will be greater in field than laboratory studies because of the challenges of assigning paternity in the wild. We then discuss why redressing this sex skew is important in terms of improving our understanding of evolutionary theory, modelling population dynamics in a changing world and developing effective conservation strategies. Finally, we outline ways to redress this skew and better use data that are already available to improve our understanding of sexual dimorphism in life histories.

## 2. Why do life histories often differ between males and females?

Male and female life histories may differ in many ways. Discussing each of these, and the underlying evolutionary and cellular mechanisms that drive these differences, exceeds the scope of this review. Moreover, a number of excellent manuscripts on these subjects have been published (e.g. [17–23]). Our aim here is to summarize key hypotheses about why males and females may mature, live and die at different tempos to illustrate why sex differences in life histories may be widespread.

### (a) The importance of asymmetric inheritance

When it comes to shaping sexually dimorphic lifespans, asymmetric transmission of genetic material seems important. Males and females inherit genetic components asymmetrically—mitochondrial DNA is maternally transmitted, and in a myriad of genetic sex-determination systems the sex chromosomes have differential residencies in each sex. This asymmetrical inheritance may have maladaptive consequences that explain sex differences in longevity. For example, the ‘unguarded X’ hypothesis posits that the heterogametic sex (males in X–Y systems and females in Z–W) will be shorter lived and experience a steeper decline in function with age because of the increased expression of deleterious recessive mutations in the sex chromosome [24]. The recessive mutations will be expressed in the heterogametic sex unconditionally, whereas these will be guarded in the homogametic sex by the presence of the second X or Z chromosome. Studies that experimentally manipulate inbreeding levels to ‘unguard’ the X chromosome in the homogametic sex show mixed results. High levels of inbreeding minimize the lifespan differences between the sexes in some studies and provide evidence to support the ‘unguarded X’ hypothesis [25,26] but not in others [27]. The more recent ‘toxic Y’ hypothesis posits that the transposable element-rich Y or W chromosome can also drive sex differences in ageing [28,29]. The epigenetics of the Y chromosome change throughout life and if its high transposable element content gets de-repressed with age it could accelerate ageing [30]. A phylogenetic-meta-analysis reported that the size of Y rather than X chromosomes correlates negatively with male survival in mammals, concluding that sex differences in mammalian lifespan are better explained by ‘toxic Y’ rather than ‘unguarded X’ [31]. Finally, the ‘mother's curse’ hypothesis posits that shorter male lifespans can be explained by the maternal transmission of the mitochondrial genome because this allows mitochondrial mutations that are detrimental to males but not females to accumulate [32,33]. This sex-specific mutation accumulation can help explain why females are longer lived in some taxa, but not the observation of longer male lifespans in taxa such as birds [34].

### (b) Sexual selection and sexually dimorphic life histories

A key factor promoting sexually dimorphic life histories is sexual selection—reproductive competition between members of the same sex and species [35–37]. Sex differences in life-history driven by sexual selection may first appear in the timing of maturity because, as noted by Darwin [38], any male which is ready to breed first has an advantage over his competitors. Females may also use rapid development as a condition dependent cue of male quality, meaning that males that develop quickly or migrate sooner may be advantaged by female choice [39]. Accordingly protandry, where males develop more rapidly than females or arrive first at breeding grounds [39], is widespread (e.g. [40]). However, sexual selection may favour slower development in males than females if, for example, delayed maturation shortens the male reproductive season and reduces the costs of male–male competition [41].

After reaching maturity, sexual selection can promote sexual dimorphism in age-dependent survival and fertility. At the population level, sexual selection may shift how males and females schedule their reproductive investment over their lives and in turn, alter how selection acts on age-dependent mortality risk. Because females need time to amass resources needed to produce offspring—for reasons rooted in anisogamy females tend to invest more in offspring than males do—they are predicted to pursue a moderate tempo and moderate return strategy of reproductive investment [17]. By contrast, males may maximize their reproductive success by investing intensely in their early reproductive effort [17,42], which can favour the evolution of shorter lives in males than females, and possibly faster or earlier ageing. Alternatively, sexual selection can mean that older males invest more in sexual signalling [43] and have greater reproductive success than young males. This can happen in cases where older males are larger [44], hold better territories [45], or have more extensive song-repertoires [46] and thus are better at securing mates. If male reproductive success increases with age, while female reproductive success remains constant or declines; sexual selection can favour the evolution of longer lives in males than females [17]. Sexual selection could also promote longer lives (and potentially slower ageing) in males than females if female choice improves overall male quality and, through doing so, increases male longevity and slows ageing. This effect could be amplified if there is sexual conflict and genes that improve male fitness reduce female fitness [47]. Finally, at an individual level, the costs of mate competition can reduce male survival and future reproductive investment, while male harassment, mate searching or mating itself may reduce female survival and fertility [48]. Conversely, female mate choice for direct benefits (e.g. nuptial gifts) could improve female survival or future fecundity.

### (c) It is not just sexual selection—natural selection matters too

Other drivers of sex differences in life histories do not involve sexual selection. For example, natural selection can promote sex differences in age-dependent mortality if the sexes differ in their extrinsic mortality risk. Extrinsic mortality that is uniform with respect to age or stage will not affect the evolution of age-dependent mortality. However, if extrinsic mortality affects particular age classes differently or has density-dependent impacts on the vital rates of surviving members of the population that are non-random with respect to age or stage, then variation in extrinsic mortality can affect selection acting on age-dependent mortality [49–51]. If these effects are sex-specific, perhaps because the sexes have different ecological niches (as in [52]) or due to different reproductive roles (e.g. if pregnancy reduces female mobility and elevates mortality risk [53]), this may lead to sex differences in mortality and fertility trajectories [54]. However, the relationship between the risk of dying due to purely environmental causes (i.e. extrinsic mortality) and the evolution of age-dependent mortality is complicated and predicting the direction of effects challenging [49–51,55].

In summary, asymmetric inheritance of genetic material may drive sexual dimorphism in life histories. Beyond this, sexual and natural selection can both promote sexual dimorphism in life-history strategies. As a result, in many species with diverse sex-determination systems, males and females often mature, live and die at different tempos, and thus, sexual dimorphism in life histories is widespread. However, life-history traits are frequently aggregated at species level for comparative analyses and conservation purposes [56,57]. While the tendency to aggregate in this way has been criticized because it obscures variation in populations in time and space [56], only brief references are often made to possible sexual dimorphism in these traits [57]. This aggregation across the sexes is however somewhat inevitable, if life-history data for males are scarce.

## 3. Is there a sex skew in life-history data?

Many authors have noted a sex skew in life-history data—more specifically, that our understanding of male reproductive scheduling is poor [10,11,15]. Here, to quantify the scale of this skew objectively and comprehensively we used the open-access Demographic Species Knowledge Index [58]. This meta-database was released in 2019 and collates demographic and life-history traits from 22 data repositories and classifies the level of demographic data for 32 144 species of mammals, birds, reptiles and amphibians [58]. This index determined whether there are data on reproductive traits available for a species (i.e. age at first reproduction, inter-litter or inter-birth interval, litter or clutch size, proportion of adult females that are reproductive or birth or recruitment rate) and if there are available mortality/survival data (i.e. maximum recorded lifespan, mean age of (adult) population and crude mortality). The database also recorded combined age or stage survival-reproduction knowledge, i.e. age- or stage-specific death and reproductive rates, mostly contained in life tables or matrix population models (MPMs). Crucially, the database recorded if these demographic data were collected in males, females, both sexes separately (i.e. male and female) or combined or whether sex was unknown.

**Figure 1.**
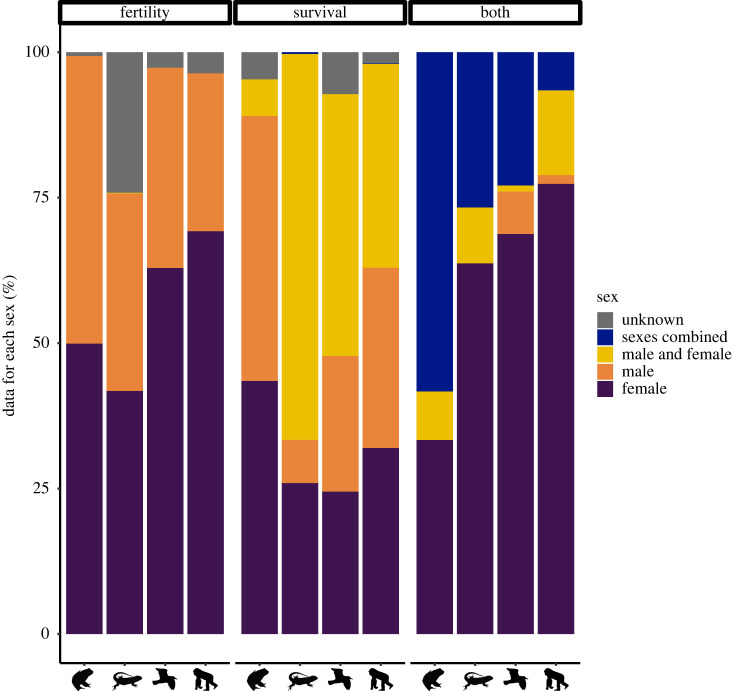
Proportion of data from each sex category for each animal class for reproduction or fertility, survival or mortality and reproduction and survival combined from data available in the Demographic Species Knowledge Index. Proportion data are calculated from the number of datasets where sex information is provided (i.e. exclude cases where sex = NA). Sex categories used include ‘female’, which means that particular dataset refers to females only, ‘male’ means that particular dataset refers to males only, ‘male and female’ means that sexes were studied separately (i.e. data are available for both sexes) and 'combined’ means that both sexes were studied together (i.e. data for the sexes cannot be decoupled). (Online version in colour.)

We determined how many datasets (i.e. rows in the database) are available for each trait and sex, while excluding datasets lacking sex information entirely (i.e. sex = NA, which is a different category than sex = unknown; electronic supplementary material, table S1). All summary statistics reported here refer to this subset of the main database i.e. datasets where sex information is provided. These data show that for survival and mortality data, the spread of data is relatively even across the sexes (figure 1 and table 1). This is not necessarily true of the data for reproduction. In amphibians, reproduction data are readily available in both sexes (49.89% female, 49.45% male). However, in mammals, 69.22% of reproduction data in the database originate from females alone and only 27.13% from males, while in birds, 62.89% of reproduction data originate from females and 34.42% from males. This is a pronounced skew, perhaps in part exacerbated because some reproductive traits featuring in the database must inevitably be collected in females i.e. the proportion of adult females that are reproductive. The skew towards female data being more abundant is particularly pronounced when considering combined age- or stage-specific survival-reproduction knowledge i.e. data from life tables or MPMs. In mammals for example, 77.36% of these data originate from females, 14.57% considers both sexes separately, and 1.51% males alone. This offers strong support for the general consensus (and our first prediction) that knowledge of how males schedule their reproductive effort over age lags far behind our understanding of female fecundity schedules. Moreover, the skew is most pronounced in mammals, followed by birds and then reptiles and apparently absent in amphibians. This broadly supports our prediction that in taxonomic groups where parental care is largely absent, the skew will be less pronounced. The rationale behind this prediction being that the relative difficulty of quantifying reproductive success in fathers relative to mothers is more pronounced in systems with prolonged maternal care (as is typical in mammals [16]) or biparental care albeit with the possibility of extra-pair paternity (common in birds [16]) than in systems where typically neither sex provides care. However, the degree of skew towards female data also correlates well with overall reproductive data availability. In the subset of the Demographic Species Knowledge Index database that we analysed, the skew is most pronounced in mammals, where reproductive information is greatest and least pronounced in amphibians, where reproductive information is lacking. The relative difficulty of assigning reproductive success to either sex in amphibians and reptiles may help explain the relatively low sex skew, but also the general lack of data availability for these taxa.

**Table 1.** Data available from the Demographic Species Knowledge Index for each animal class and sex. Numbers represent total sample sizes and percentages are shown in parenthesis. Note that these are counts of datasets meaning that the same species may be represented in multiple entries. Moreover, datasets in the database where sex = NA are not shown here. Sex categories used include ‘female’, which means that a particular dataset refers to females only, ‘male’ means that particular dataset refers to males only, ‘male and female’ means that sexes were studied separately (i.e. data are available for both sexes) and ‘combined’ means that both sexes were studied together (i.e. data for the sexes cannot be decoupled).

class	sex	reproduction or fertility	survival or mortality	reproduction, survival combined
Amphibia	female	227 (49.89)	297 (43.48)	8 (33.33)
	male	225 (49.45)	311 (45.53)	0 (0)
	male and female	0 (0)	43 (6.3)	2 (8.33)
	sexes combined	0 (0)	0 (0)	14 (58.33)
	unknown	3 (0.66)	32 (4.69)	0 (0)
Reptilia	female	1140 (41.76)	1104 (25.93)	186 (63.7)
	male	927 (33.96)	316 (7.42)	0 (0)
	male and female	3 (0.11)	2823 (66.31)	28 (9.59)
	sexes combined	0 (0)	14 (0.33)	78 (26.71)
	unknown	660 (24.18)	0 (0)	0 (0)
Aves	female	2849 (62.89)	546 (24.46)	396 (68.75)
	male	1559 (34.42)	520 (23.3)	42 (7.29)
	male and female	0 (0)	1005 (45.03)	6 (1.04)
	sexes combined	0 (0)	0 (0)	132 (22.92)
	unknown	122 (2.69)	161 (7.21)	0 (0)
Mammalia	female	4695 (69.22)	457 (31.96)	1179 (77.36)
	male	1840 (27.13)	443 (30.98)	23 (1.51)
	male and female	0 (0)	501 (35.03)	222 (14.57)
	sexes combined	0 (0)	1 (0.07)	100 (6.56)
	unknown	248 (3.66)	28 (1.96)	0 (0)

## 4. Why do we lack data on male reproductive success?

Every individual in a sexually reproducing species has one father and one mother—so, we know on average what male reproductive success looks like, but clearly we do not know how much this success is skewed towards particular males, or how reproductive success varies over age. This is because while measuring age-dependent reproductive success in females is relatively straightforward—counting offspring hatched, weaned or fledged—measuring male reproductive success is complicated. Simply, male reproductive success must be measured via females. While females tend to invest heavily in material contributions to the next generation (e.g. investment in large ova, gestation and lactation), males invest less in such material contributions and, instead, raise their reproductive effort to the optimum by investing in other traits [59]. These traits may include contests (e.g. fights or sperm competition), mate searching, elaborate displays to attract females, courtship during copulation or some other engagement with cryptic female processes, as well as transferring enough functional sperm to successfully fertilize ova [36,60,61]. Measuring male reproductive success in a biologically meaningful way therefore requires quantifying paternity outcomes against a background of male–male competition and mate choice scenarios—this is not easy. In the laboratory, one approach is to measure male siring success with multiple females in competitive mating assays (i.e. measuring male reproductive success in the presence of competing males). To capture post-copulatory elements of male reproductive success (e.g. sperm competition and cryptic female choice), researchers must be able to assign paternity in multiply mated females or otherwise measure ejaculate investment and costs of copulatory courtship. Thus, measuring male siring success via a combination of competitive and non-competitive assays (e.g. [62]) would be the gold standard in a laboratory environment but this is a labour intensive approach that requires large numbers of animals. In the field, biologically meaningful estimates of male reproductive success may be easier to quantify because ecologically relevant male–male competition and female choice before and after mating are in action in wild populations. However, it is difficult to assign paternity in the wild, especially if polyandry is common, meaning that DNA samples are needed from offspring and potential fathers to correctly assign paternity, which may not be feasible for some study organisms and systems.

Given this complexity, it is not surprising that we lack data on age-dependent male reproductive success. It is also not surprising that existing measures of male reproduction often use single measures of male reproductive investment (e.g. calling effort in crickets [63], discussed further below) rather than direct measures of male reproductive success. While measuring reproductive success in males is challenging, we suggest that there are evolutionary ecological insights that may be gleaned by collecting these data and more generally, ensuring that life-history data from both sexes are available.

## 5. Do missing males matter?

As highlighted previously, life histories can be sexually dimorphic but we lack data on male reproductive investment over age, and life-history data are often aggregated at species level for conservation purposes or comparative analyses [56,57]. Here, we suggest reasons why better accounting for sexual dimorphism in life-history traits is important, from both theoretical and applied perspectives.

### (a) The sex bias in life-history data means that we cannot test some hypotheses

The antagonistic pleiotropy theory of ageing proposes that because natural selection weakens over age, alleles with positive effects on early-life fitness are favoured by selection even if they have negative, pleiotropic effects expressed late in life [54]. These pleiotropic late-acting effects could cause ageing. The author of this hypothesis, George Williams, proposed that that one way to test his theory was to characterize sex differences in ageing rates, stating that ‘where there is a sex difference (in ageing), the sex with the higher mortality rate and lesser rate of increase in fecundity should undergo the more rapid senescence’ [54]. However, the strong skew towards females in the Demographic Species Knowledge Index for age- or stage-specific survival and fecundity information (table 1) suggests that we lack the data needed to test this hypothesis.

**Figure 2.**
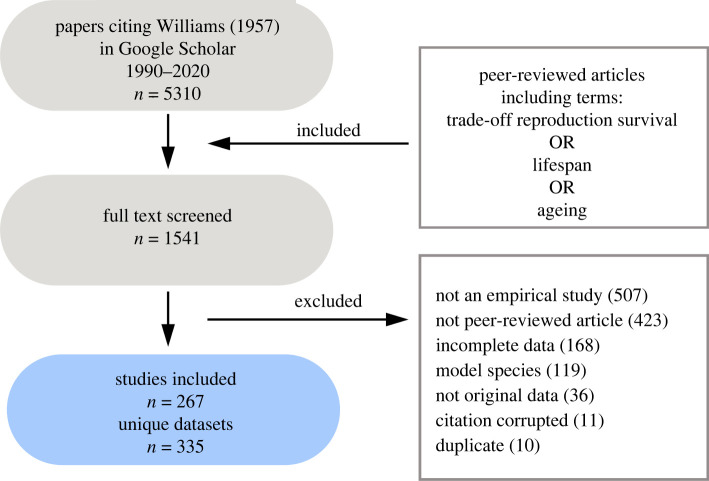
Prisma diagram outlining our antagonistic pleiotropy search procedure. More detail is provided in the electronic supplementary material, text S1. (Online version in colour.)

**Figure 3.**
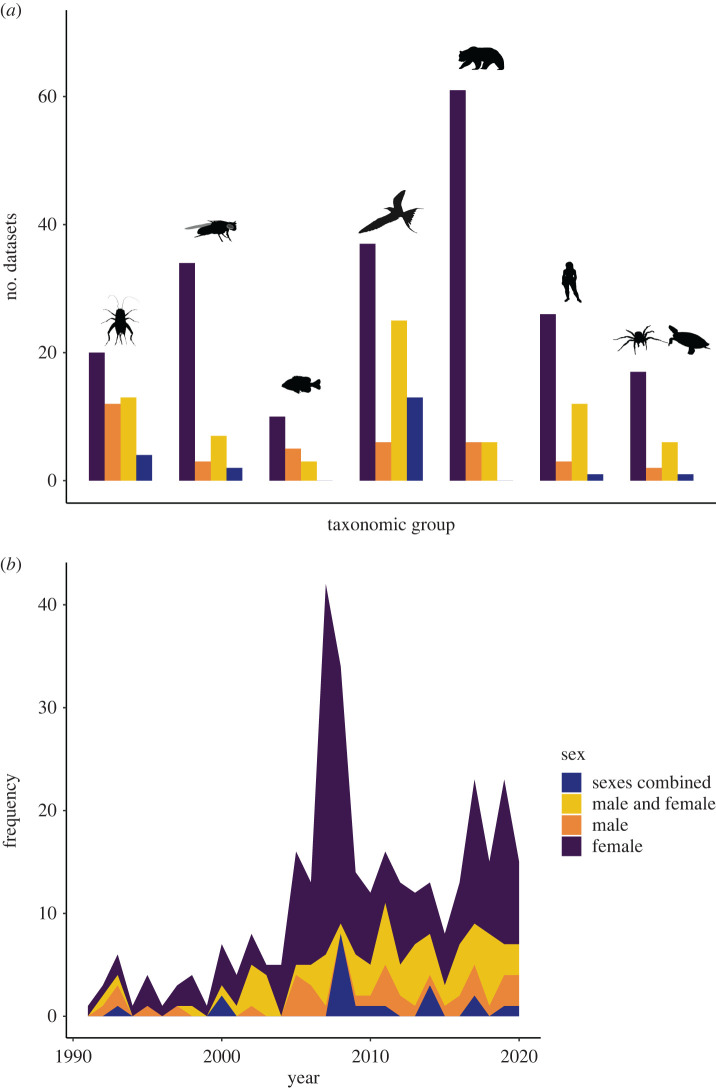
(*a*) Number of datasets for each taxonomic group (insects, *Drosophila melanogaster*, fish, birds, mammals, humans and others) and sex category, and (*b*) timeline of numbers of published datasets split by sex categoury. *D. melanogaster* and humans have shown separately because these species featured heavily in the search output. (Online version in colour.)

More generally, Williams predicted a trade-off between early and late life fitness. A logical extension of this given his reference to sex differences in reproductive scheduling is that males and females may resolve this trade-off differently. And yet, literature testing how early reproductive investment trade-offs against future survival and reproduction appears to focus on females. To gain some insight into if this is the case, we searched for manuscripts citing Williams's original antagonistic pleiotropy theory published between 1990 and 2020, that include the terms (‘trade-off’ reproduction survival OR lifespan OR ageing) in Google Scholar (figure 2). From this search, we identified peer-reviewed research papers that measured a reproductive trait at multiple ages or recorded a measure of reproductive investment and a measure of survival (e.g. lifespan and age-dependent mortality risk) (details in electronic supplementary material, text S1). We found that data were biased firmly towards females—with 2.5 × more data being available for females than males overall (figure 3*a*; electronic supplementary material, table S2). Once more the magnitude of this skew varied between taxa. However, in contrast with our prediction that the skew may be less pronounced in taxonomic groups where parental care is rare, in some taxa where care is largely absent (e.g. reptiles) no male data were available at all (electronic supplementary material, table S3), and in others, data were still heavily skewed towards females (e.g. insects). Moreover, and in contrast with our earlier prediction, this bias appears to be slightly more pronounced in laboratory studies—in the field 36.2% of studies collect data for males independently of females, while this is only true of 27.9% of studies in the laboratory (electronic supplementary material, figure S1 and table S4). We also quantified whether these studies measured reproduction directly (e.g. egg or offspring counts) or indirectly (e.g. proxies for reproductive investment such as sexual signalling). 96.4% of the available data reported some direct measure for females, while only 59.6% of data reported direct measures of male reproductive success (electronic supplementary material, table S5). Crucially, however, males appear to feature more frequently in more recent manuscripts (figure 3*b*), meaning that while our understanding of trade-offs involving reproduction is biased towards females overall, there are signs that this bias is being addressed.

In summary, data from the Demographic Species Knowledge Index confirm that 70 years after Williams made his prediction about sex differences in fecundity scheduling translating to sex differences in actuarial senescence, we lack the data to test this prediction on a large scale [11]. Further, our semi-quantitative review suggests that our understanding of how the sexes trade-off early and late reproduction or reproduction and survival is biased towards females—this hinders efforts to compare how the sexes manage trade-offs involving the costs of reproduction more generally.

### (b) The sex skew in data can lead to patterns emerging (or being obscured) that do not accurately reflect species' biology

Inaccurate inference because of sex skew is demonstrated by a meta-analysis [15], which synthesized dietary restriction effects on the expression of reproductive traits. Only 21% of the effect sizes extracted involved males, and of these, less than 2% used measures of male reproductive investment that captured a major portion of costly male reproductive traits (e.g. male–male competition, mate attraction and fertilization success) [15]. By contrast, 48% of the female effect sizes captured a large portion of female reproductive investment [15]. This bias affected the outcomes of the analyses. Initially, there was a sex difference in how reproduction responded to dietary restriction but when the analyses incorporated whether the traits measured captured a small, intermediate or large portion of sex-specific reproductive costs, this sex difference disappeared. This suggests that apparent sex differences in how reproduction responds to dietary restriction may be an artefact of a failure to accurately measure how diet affects male reproductive investment.

Similarly, in research testing the mechanisms regulating life histories, it is vital that both sexes are studied to generate robust conclusions. This is because the mechanisms that shape life histories, and how they are affected by the environment, may differ between the sexes. This is demonstrated by research exploring the evolutionarily conserved signalling pathways, target for rapamycin and insulin/insulin-like growth factor 1. While some genes in these pathways respond to particular dietary manipulations in the same way across the sexes, sets of genes linked to reproduction display opposite expression patterns in each sex, suggesting that the sexes diverge in how nutritional information is translated into reproductive regulation [64]. This means that testing theories regarding the mechanistic basis of life histories in one sex alone may generate conclusions that do not apply to both sexes equally.

More generally, in comparative analyses, life-history data are aggregated at species level (e.g. [13,57]). If males and females frequently trade-off life-history traits differently as predicted by theory [17] and already observed in some species [65], outcomes of analyses are likely to differ to some degree when the sexes are considered separately. Accordingly, drawing more concrete conclusions about how species resolve life-history trade-offs, and better characterizing an important aspect of biological diversity, requires improved integration of male demographic data into comparative analyses.

### (c) Better understanding the sex-specific responses to particular treatments or interventions can help us to improve the health of our own species

Some interventions aimed at improving longevity have sex-specific impacts on phenotype. The Interventions Testing Program investigates the potential of drugs and supplements delivered to mice via their diet to promote healthy ageing [66]. The programme showed that while glycine and rapamycin treatment increased lifespan in males and females, nordihydroguaiaretic acid, protandim, aspirin and 17-α-oestradiol increase lifespan in males but not females [67–71]. This highlights the need to consider the sexes separately for biomedical applications, because results obtained from females are poor predictors of male responses in anti-ageing drug discovery trials (and *vice versa* [72]). Similarly, it is imperative to understand how these treatments modify age-dependent reproductive investment in both sexes if given early in life. It is important to flag, however, that historically males are more frequently used as animal models of disease [72]. While this bias is less prevalent in recent research, none-the-less for biomedical applications the sexes are frequently analysed together, and analyses neglect to test for, or report, explicit sex differences in outcomes [73].

### (d) Studying both sexes can improve our ability to predict how species will respond to environmental change

Characterizing vital rates (survival and fertility) in populations is key to understanding population responses to environmental change. There are many reasons why it is important to collect these data in both sexes. First, one sex may demonstrate signals of reduced fitness in response to a changing environment before the other. For example, in many insects sperm function is reduced at high temperatures, impacting male fertility [74], but these effects may not be detected if only female reproductive success is assayed because females can mitigate these effects to a degree by multiple mating [75]. Here, reduced fertility in males offers an early warning signal that rising temperatures might lower overall population productivity if females cannot keep buffering the effects of reduced male fertility via multiple mating.

A second example is provided by a rookery of the endangered green turtle (*Chelonia mydas*)—a species with temperature-dependent sex determination. In this rookery, offspring sex ratios are highly female biased, a situation exacerbated by climate change; however, adult sex ratios on breeding grounds are male biased. This seems to represent more frequent mating activity by males. Thus male mating behaviours may buffer the population from the deleterious impacts of climate change [76]. However, understanding the skew in male reproductive success in this system offers insight into its effective population size, which is an important parameter in terms of predicting long-term population persistence [76].

More generally, differences between female and male demography can affect the reproductive output of a population under environmental change [77] and promote selective harvesting of males via hunting, thus altering population structure and evolutionary life-history trajectories [78]. Sex differences in reproductive behaviours within local populations may be coupled with sex-biased dispersal [79,80], and understanding this bias has been important to understanding invasion [81] and modelling population dynamics [82]. Accordingly, incorporating both sexes into population dynamic models may improve their predictive power and thus, help develop more effective conservation strategies [10].

## 6. Future steps

Many long-running field projects have collected demographic data in both sexes over years or even decades (e.g. [83–85]) and open-access databases facilitating large-scale demographic analyses endeavour to record data separately across the sexes where possible (e.g. COMADRE [86]). Moreover, our search of the antagonistic pleiotropy literature suggests male data on reproductive investment are being collected more frequently now than previously. So, perhaps the sex skew in our understanding of male reproductive scheduling is on route to being remedied. However, in the short term, what can we do to improve our understanding of male reproductive scheduling given the challenges of measuring male reproductive success?

First, while much existing data on male reproductive success appears to rely heavily on proxy measures (e.g. investment in sexual display traits such as pigmentation or weaponry) rather than measuring male reproductive success *per se* (electronic supplementary material, table S5), reduced costs of sequencing mean that paternity analyses is an increasingly accessible means of directly measuring male reproductive success. Using such approaches to measure male reproductive success directly would be positive. As would applying these techniques to demonstrate that proxy measures of reproductive success that are easier to measure are appropriate and consistently correlate well with male reproductive output.

Further, there is potential to make some data from population models more accessible for the purposes of comparative analyses of life histories. For example, many of the demographic data available in open-access repositories used for comparative analyses are stored in the form of life tables, or age- or stage-structured population models (e.g. [87]). Many tools are available to analytically obtain life-history traits from these types of structured demographic data, even if the original publications did not report such traits [88,89]. Age- or stage-structured models are best suited for easily observable components of populations, in species lacking complex interactions among individuals of either sex. Therefore, sex differences in life-history processes are mostly accounted for in models that explicitly consider individual breeding or movement patterns. These models tend to be parametrized as agent-based models. For instance, individual-based models (IBMs) have investigated how sex-specific parasitic infections can induce sex-specific dispersal strategies in a metapopulation [90] and how complex interactions across the sexes affect population dynamics [91]. IBMs have also been used to assess the optimal size of sex change in hermaphroditic species [92] and the demographic consequences of such change [93]. With improved data availability and sophisticated modelling tools, these more mechanistic approaches are increasingly being used [94], but unless they directly report ‘classic’ life-history traits, such traits cannot be obtained analytically from modelling outputs and thus, these studies are omitted from many global databases. Deriving life-history information from IBM outputs and integrating this information into databases would be one way of increasing the data available for large-scale comparative analyses of male and female life histories.

In the absence of detailed data on sex-specific demography, a first step towards integrating sex differences into comparative analyses may consist of obtaining information on sex ratios across a wide range of taxa. Analysing sex ratios has a long history in evolutionary demography [95], and sex ratios can be considered a key component of life-history evolution [96,97]. However, sex ratios as a life-history trait have largely been omitted from comparative studies thus far [13,98–100] (but see [101]). One main argument for this has been that females (not males) typically limit reproductive output. However, the most limiting sex may differ over time [102,103]. In fact, feedback between differences in male and female reproductive investment and changes in population structure, produce variation in the sex ratios of a population. Thus, integrating sex ratios into comparative life-history analyses could provide new insights into an additional axis of life-history variation, and one which has important population demographic consequences. Such integration will only be possible if sex ratios are incorporated into online demographic databases, which is currently not standard practice [58], but is at least provided as supplementary information is some databases [86].

## 7. Conclusion

While theoretical work has long acknowledged differences in male versus female life-history strategies and the effect that this may have on population dynamics, empirical work on reproductive scheduling still largely focuses on females. While there is variation across taxa—the skew being particularly pronounced in mammals and birds—the bias is evident in taxonomic groups where mothers provide the majority of parental care and in taxa where care is absent. Moreover, the bias is evident in both field and laboratory studies. Even where male reproduction is quantified, indirect measures of reproduction are frequently used rather than direct measures of male reproductive success. Crucially, however, male data are being collected more frequently and this opens up the possibility of tackling some long-neglected research questions and improving the power of demographic forecasting. Additionally, recent theoretical and methodological advances may help rectify the sex skew. For example, advances in population ecology that use mechanistic modelling approaches to incorporate complex sex-specific mating and movement patterns into assessments of population persistence can facilitate the integration of male life histories into comparative analyses. This is encouraging and should incentivise laboratory studies to invest into research that can help parameterize these mechanistic models using explicit inheritance or selection information. Additionally, advances in animal tagging methodologies (e.g. miniaturization of GPS tags, methods for better distinguishing between GPS tag failure and animal death [104]) make it increasingly possible to collect demographic data in the field. Reduced costs mean that next-generation sequencing is increasingly accessible as a means of assigning paternity (but see [105]) and new tools are being developed to analyse paternity from single-nucleotide polymorphic markers [106]. These approaches may help us understand why the sexes live, reproduce and die at different tempos, and these data may have applied impacts for the health of managed populations and predicting population responses to environmental change. Until then, while male fecundity data lags behind female fecundity data, it is important to acknowledge the potential impact sexual dimorphism may have on the conclusions of analyses of datasets that are heavily female biased or, where demographic data are aggregated across the sexes.

## Data Availability

Data cited in this review and code are available at https://osf.io/r6tce/. Additional results and methods information areprovided in the electronic supplementary material [107].
